# Motor Cortex Function in Fibromyalgia: A Study by Functional Near-Infrared Spectroscopy

**DOI:** 10.1155/2019/2623161

**Published:** 2019-01-16

**Authors:** Eleonora Gentile, Katia Ricci, Marianna Delussi, Filippo Brighina, Marina de Tommaso

**Affiliations:** ^1^Applied Neurophysiology and Pain Unit, SMBNOS Department, Bari Aldo Moro University, Polyclinic General Hospital, Via Amendola 207 A, 70123 Bari, Italy; ^2^Department of Experimental Biomedicine and Clinical Neurosciences (BioNeC), University of Palermo, Via del Vespro 143, 90127 Palermo, Italy

## Abstract

Previous studies indicated changes of motor cortex excitability in fibromyalgia (FM) patients and the positive results of transcranial stimulation techniques. The present study aimed to explore the metabolism of motor cortex in FM patients, in resting state and during slow and fast finger tapping, using functional Near-Infrared Spectroscopy (fNIRS), an optical method which detects in real time the metabolism changes in the cortical tissue. We studied 24 FM patients and 24 healthy subjects. We found a significant slowness of motor speed in FM patients compared to controls. During resting state and slow movement conditions, the metabolism of the motor areas was similar between groups. The oxyhemoglobin concentrations were significantly lower in patients than in control group during the fast movement task. This abnormality was independent from FM severity and duration. The activation of motor cortex areas is dysfunctional in FM patients, thus supporting the rationale for the therapeutic role of motor cortex modulation in this disabling disorder.

## 1. Background

Fibromyalgia (FM) is a disabling disease with widespread muscle pain, associated with fatigue, sleep disorders, cognitive impairment, and a number of other physic and psychopathological symptoms [1]. It is a complex and often poorly curable syndrome, with unclear pathophysiological mechanisms. Most experimental evidence suggests a central failure in pain modulation with abnormal response to nociceptive stimuli [2–4]. Increasing evidence is in favor of the pivotal role of the motor function in contributing to pain syndrome outcomes [5]. As a matter of fact, patients with chronic pain have deficits in motor performances, with compromised coordination and control force [6, 7]. However, definite data about the functional condition of motor cortex in FM patients is still lacking. Cortical excitability characteristics are not constant in time, but they depend upon clinical status. In addition, different neurophysiological methods could give different results [8]. Meta-analyses provided evidence of increased M1 long-interval intracortical inhibition in chronic pain [5]. The possible basal condition of motor cortex inhibition and the benefit of its recovery in pain control may explain the often-reported success of excitatory neuromodulation of M1 in patients with fibromyalgia [8–10].

Brain hemodynamic activity is considered a good measure of cortical activation during the performance of a motor task. In neuroimaging studies the finger tapping task is widely used to investigate the function of the motor cortex [11–13].

Functional Near-Infrared Spectroscopy (fNIRS) is a noninvasive technique that allows a real time detection of blood flow and metabolism changes in the cerebral cortical tissue. To measure functional brain activity, fNIRS employs emitters, named sources, that release optical radiation within head tissue detected by scalp detectors. Thanks to its low cost, good temporal resolution, portability, and movement tolerance, the fNIRS is a useful tool in experimental neuroimaging studies on motor functions [14].

In the present study we aimed to apply the fNIRS method to detect the basal status of motor cortex and its changes under motor activation in FM patients compared to controls. We used 2 types of motor tasks, a finger tapping task at fixed intervals and a finger tapping task at the maximal individual velocity, to establish if a possible cortical metabolic dysfunction was present in basal condition and/or during simple movement and/or during maximal motor performance.

The specific purposes of this pilot study were as follows:

(1) To evaluate oxyhemoglobin concentrations of motor cortex in resting state in FM patients and controls

(2) To detect fNIRS changes under slow finger tapping task

(3) To detect fNIRS changes during finger tapping speed test and correlate it to motor performance

(4) To correlate fNIRS and clinical data in FM patients

## 2. Materials and Method

The study included consecutive outpatients who came for the first time to our Unit in the period between January and June 2017. The selection criterion was the diagnosis of fibromyalgia according to the ACR criteria [1]. Controls were selected among hospital staff. Exclusion criteria were less than 8 years of education; any peripheral or central nervous system (CNS) disease, including spinal cord diseases and radiculopathies; psychiatric disease; diabetes; active and/or positive history for thyroid insufficiency; renal failure; autoimmune diseases; inflammatory arthritis; systemic connective tissue disease; present or previous history of cancer; and use of drugs acting on the CNS or chronic opioid therapy. All the participants were right handed, as confirmed by Edinburgh Handedness Inventory [15]. The patients group included 20 females and 4 males patients (M_age_= 42,17 SD= 10,05; age range from 23 to 59 years), the control group included 19 female and 5 male control subjects (M_age_= 40,00 SD= 14,64; age range from 22 to 60 years). The experimental research received the approval of the Ethics Committee of the Bari Policlinic General Hospital. All the participants signed a written informed consent.

### 2.1. Clinical Examination

The neurologist submitted all patients to the standard neurological examination, including thorough bedside sensory testing. In accordance with recent studies [16, 17], the FM patients filled out the fibromyalgia-linked invalidity questionnaire-FIQ [18] and the Multidimensional Assessment of Fatigue-MAF [19]. Patients received indications on the modalities of the responses by a psychologist. In view of the correlation between fNIRS and clinical data, we considered the Wide Pain Index (WPI) according to the recent ACR diagnostic criteria (2010) [1].

### 2.2. Experimental Study Design

The participants sat down on a comfortable chair in a quiet room. The researcher trained the participants how to perform the finger tapping task and the resting state. The motor task consisted of pressing a toggle button with the thumb of the right hand. We evaluated the motor cortex activation during the finger tapping tasks and the speed test.

The experiment consisted of 3 conditions:

- 2 minutes of resting state, during which the participant was requested to stay relaxed with open eyes, fixing a point on the computer monitor
- Slow finger tapping task: the participant pressed the button with the right thumb every 5 seconds for 10 times
- Fast finger tapping task: the participant pressed the button with the right thumb as fast as possible

The order of experimental sessions was randomized and the pretask baseline was 1 minute in duration.

### 2.3. FNIRS Procedure

The hemodynamic activity was recorded by fNIRS compatible cap. We used the fNIRSport instrument (Wearable fNIRS Imaging System) to detect the fNIRS signal. To acquire oxy-hemoglobin concentration changes, we employed fNIRStar 14.2 Acquisition Software (Version 14, Revision 2, Release Build, 2016-04-15 (c) NIRx Medizintechnik GmbH, Berlin, Germany; https://www.nirx.net.

**Figure 1:**
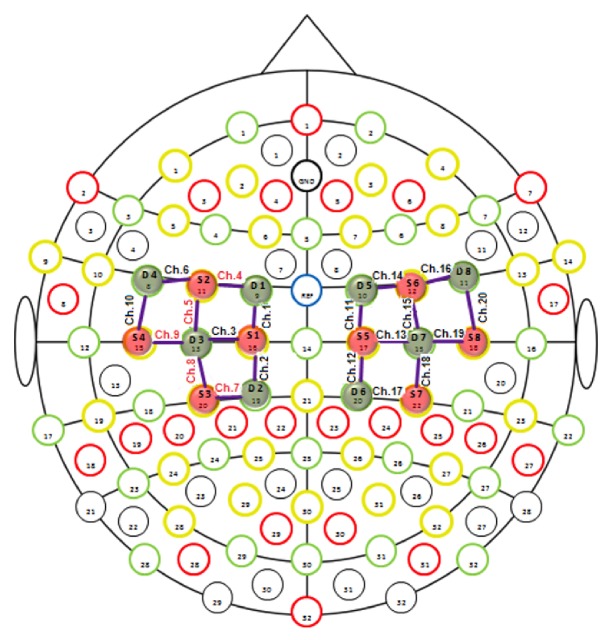
Channels and optodes configuration. In this figure the lines in purple color are the recording channels. Red circles are the sources and green circles are the detectors.

We positioned the fNIRS optodes (sources and detectors) on the cortical regions of interest, which was on the motor cortex, according to the predefined montages tab by fNIRStar 14.2 (Figure 1).

The fNIRS recording channel consists of a source that emits the near-infrared light, which spreads through the biological tissue and is received by the detectors. The distances between sources and detectors were 30 mm. The sampling rate of fNIRS signal was 7.81 Hz. The variations in oxygenated hemoglobin concentration were measured in mmol/L. For the present experiment we used 20 recording channels, 10 for each side of motor cortex, consisting of 8 sources and 8 detectors communicating with each other. Before placing the optodes on the scalp, the technician freed the surface of the skin from the hair, in order to advance the passage of the optical radiation through the brain tissue. We also positioned a covering black cap, to avoid the interference of environmental light. The technician carried out a signal quality check by quick automated calibration using the signal acquisition software. Before proceeding with the recording, the experimenter ascertained the good quality of the fNIRS signal.

### 2.4. FNIRS Analysis

We used the NIRSlab Software (v2014.12; NIRx Medical Technologies LLC) to process raw data. The raw fNIRS signal was digitally bandpass-filtered offline at 0.01-0.3 Hz. Moreover, the optical density data were converted into relative oxyhemoglobin concentration variations, according to modified Beer-Lambert Law [20]. To evaluate the cerebral regions of interest, we considered the oxyhemoglobin concentration variation, in accordance with several experimental studies, which indicate this parameter as the most sensitive for the assessment of the cortical metabolism during finger tapping task execution [21, 22]. We thus considered the difference between the mean values of oxyhemoglobin concentrations in the resting state and finger tapping tasks, divided for the standard deviation of mean baseline values [23]. For each subject and each recording channel, the effect size (Cohen's d) was calculated.

The speed during fast finger tapping task was calculated through the number of clicks per second by manual computation.

### 2.5. Statistical Analysis

The IBM SPSS Statistics software, version 21, was employed. The values of oxyhemoglobin concentration in resting state, slow and fast movement finger tapping tasks were compared among groups, considering the channels as variable, with a MANOVA test, complete factorial type III model. We merged the mean oxyhemoglobin levels of the significant channels into a Region of Interest (ROI). The finger tapping speed was compared between groups by one way ANOVA. The ROI values were correlated with the finger tapping speed using the linear regression test in FM and control groups. They were also correlated with age and clinical features by Spearman correlation test. We considered for each statistical analysis p < 0.05 as significant.

## 3. Results

### 3.1. Finger Tapping Speed

**Figure 2:**
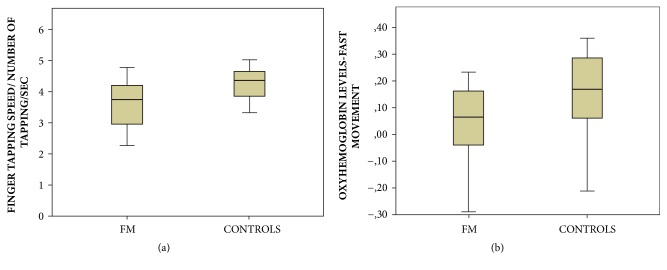
(a) Mean values and standard deviation of speed values in fibromyalgia patients (FM) and controls. One way ANOVA F 10 DF 1 p 0.003. (b) Mean values and standard deviation of oxyhemoglobin levels change on the ROI –CH4,5,7,8,9 during fast movement in fibromyalgia patients (FM) and controls. One way ANOVA F 6.44 DF 1 p 0.015.

Movement was significantly slower in FM patients, compared to healthy controls (Figure 2(a)).

### 3.2. Oxyhemoglobin Levels

#### 3.2.1. Resting State

In resting state the oxyhemoglobin concentration was similar between FM patients and controls (MANOVA F –Roy Square 0.45, Degree of Freedom Hypothesis (H-DF) 20; error DF 27, p 0.96).

#### 3.2.2. Slow Finger Tapping Task

Oxyhemoglobin changes occurring during slow finger tapping task were similar between patients and controls (MANOVA: F -Roy Square- 1.2, hypothesis DF 20, error DF 27 p 0.31).

#### 3.2.3. Fast Finger Tapping Task

**Figure 3:**
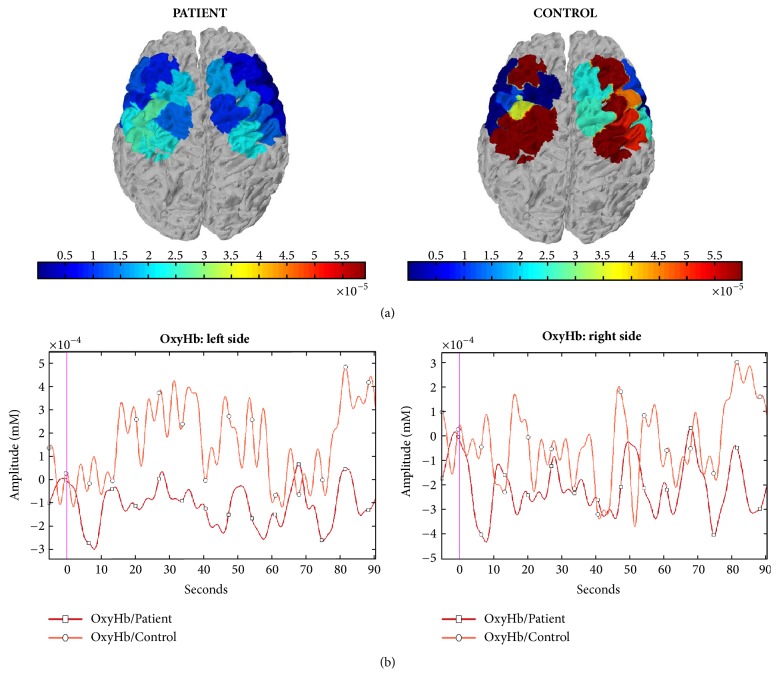
(a) Hemodynamic changes in a FM patient (female, 46 years old) and a control subject (female, 47 years old) during fast movement condition according to statistical parametric mapping methods. (b) Raw oxygenated hemoglobin levels during fast movement in the same cases. The Grand Average data of right and left channels are shown.

The oxyhemoglobin levels were lower in FM groups during the fast finger tapping task, at least on Ch-4,5,7,8,9 corresponding to the left side (MANOVA: F –Roy Square – 1.08; hypothesis DF 20, error DF 27 p 0.41, single p values; Ch-4 0.010; Ch-5 0.043; Ch-7 0.027; Ch-8 0.030; Ch-9 0.034). We thus averaged channels values into a unique ROI value (Figures 2(b) and 3).

#### 3.2.4. Correlations

The linear regression analysis between motor speed and oxyhemoglobin changes during fast finger tapping task, detected on the ROI, was not significant either in patients or in controls (FM patients: beta 1.12 p 0.55; control: beta 0.17 p 0.41).

No correlation was present between oxygenated hemoglobin and illness duration (Spearman test -0.178 n.s.), disability linked to fibromyalgia (-0.102 n.s.), WPI score (-0.180 n.s.), and fatigue ( -0.192 n.s.). No correlation emerged between finger tapping speed and the same clinical variables, excluding fatigue, which was more severe in slower patients (Spearman test -0.466 p 0.011).

## 4. Discussion

In the present study we evaluated motor cortex function using functional Near-Infrared Spectroscopy in FM patients compared to control subjects.

Main results consisted of normal oxyhemoglobin levels in resting state and during slow finger tapping task in FM patients, while patients showed lower metabolic activation on the left hemisphere during fast finger tapping task. This was not linearly correlated with the motor slowness patients exhibited in comparison to controls nor with clinical features. In the next paragraph, these results will be discussed in detail.

Recent studies have shown that the baseline characteristics of M1 are altered in patients with fibromyalgia [5–7] and that activity patterns in response to experimentally induced pain are abnormally enhanced [4, 24]. Studies using TMS methods and assessing cortical silent period and intracortical inhibitions found a reduction of inhibitory activity and increase of network excitability in cortical motor regions [5, 25]. The fMRI studies showed that during acute painful stimulation, FM patients showed increased M1 activation, a sign of a dysfunctional behavior aiming at exerting a full inhibitory control over the pain-related neural circuits [24].

Our fNIRS results were not in agreement with a basal dysfunction of motor cortex in FM patients, as the oxyhemoglobin levels were quite similar to controls. However, the oxygenated hemoglobin concentration, used to detect changes in cerebral hemodynamics, has no absolute values, so it could be quite variable across subjects. [26]. The abnormal function of motor cortex could thus appear under specific procedures of activation. During slow finger tapping task, we did not observe a different level of oxyhemoglobin change in patients compared to controls, while this emerged during the speed task.

The regions we identified as critical for the metabolic changes induced by the right-hand movement corresponded to the left prefrontal regions. The primary and probably supplementary motor cortex related to the moving hand had a reduced activation in patients. This result may be in apparent disagreement with the hyper excitability of motor networks emerging in FM patients in TMS experiments [24]. However, this paradoxical hyper excitability may be a compensatory phenomenon to a basal dysfunctional performance of motor networks [8]. Changes of cortical excitability may result in complex changes of cerebral blood flow and oxygenated hemoglobin [27]. The reduced motor cortex metabolism may thus be a final result of the prolonged activation of pain-related circuits, with a chronic inhibition of motor network and paradoxical increase of excitatory transmission [25]. The speed test revealed a reduced motor performance in FM patients. Fibromyalgia patients are affected by slowness in motor initiation [28, 29]. A study on fNIRS-related finger tapping changes in FM patients confirmed reduced motor performances and reduced activation of several brain areas that showed enhanced oxygenated hemoglobin levels in controls [30]. Our study cannot resolve the question of whether the motor impairment could be a consequence or a cause of cortical motor network dysfunction. Motor speed in the finger tapping task and oxyhemoglobin changes of motor cortex were not linearly correlated, either in patients or in controls. In line with the results of previous studies [31] we did not find a significant relationship between the finger tapping speed and the levels of cortical activation. In any case, in FM patients motor cortex dysfunction seemed to emerge during energy-demanding tasks, while oxygenated hemoglobin was normal during the slow movement and in the resting condition. The metabolic deficit emerging during fast finger tapping task seems a basal feature of FM, not correlated with disease duration and severity.

This means that it could not be a consequence of the prolonged inhibition exerted by the pain circuits activation, but a basal condition able to facilitate pain maintenance. Following this line of thinking, cortical motor dysfunction and movement impairment could characterize FM at its onset [32]. In the present FM sample, fatigue, which is a fundamental symptom of the disease, [1] was correlated with motor slowness, but not with oxygenated hemoglobin during fast finger tapping task. This could further confirm that motor failure is not in direct dependence with the motor circuits functional status, but other factors could cooperate as psychopathologic traits [33], which consideration was beyond the aim of the present study.

## 5. Conclusion

Overall, the activation of motor cortex areas during fast movement is dysfunctional in FM patients [34, 35]. These data could further support the role of movement [36], M1 transcranial modulation (particularly with new stimulation approaches as focal tDCS and tRNS) [37, 38] and even combination of both approaches [39] in the treatment of this disabling disease.

## Data Availability

The fNIRS data used to support the findings of this study are restricted by the Ethics Committee of the Bari Policlinic General Hospital in order to protect patient privacy.
